# Growth Mechanisms and the Effects of Deposition Parameters on the Structure and Properties of High Entropy Film by Magnetron Sputtering

**DOI:** 10.3390/ma12183008

**Published:** 2019-09-17

**Authors:** Yanxia Liang, Peipei Wang, Yufei Wang, Yijia Dai, Zhaoyi Hu, Denis E. Tranca, Radu Hristu, Stefan G. Stanciu, Antonela Toma, George A. Stanciu, Xingjun Wang, Engang Fu

**Affiliations:** 1 State Key Laboratory of Nuclear Physics and Technology, School of Physics, Peking University, Beijing 100871, China; yxliang@pku.edu.cn (Y.L.); ppwang@pku.edu.cn (P.W.); yfwang1@pku.edu.cn (Y.W.); daiyijia02@gmail.com (Y.D.); zyhu1@pku.edu.cn (Z.H.); 2 Center for Microscopy-Microanalysis and Information Processing, University Politehnica of Bucharest, 060042 Bucharest, Romania; denis_emanuil.tranca@upb.ro (D.E.T.); hristu_radu@yahoo.com (R.H.); sgstanciu@yahoo.com (S.G.S.); antonela2222@yahoo.com (A.T.); 3 State Key Laboratory of Advanced Optical Communication Systems and Networks, Peking University, Beijing 100871, China

**Keywords:** high entropy film, nanocrystal, deposition mechanism, magnetron sputtering

## Abstract

Despite intense research on high entropy films, the mechanism of film growth and the influence of key factors remain incompletely understood. In this study, high entropy films consisting of five elements (FeCoNiCrAl) with columnar and nanometer-scale grains were prepared by magnetron sputtering. The high entropy film growth mechanism, including the formation of the amorphous domain, equiaxial nanocrystalline structure and columnar crystal was clarified by analyzing the microstructure in detail. Besides, the impacts of the important deposition parameters including the substrate temperature, the powder loaded in the target, and the crystal orientation of the substrate on the grain size and morphology, phase structure, crystallinity and elemental uniformity were revealed. The mechanical properties of high entropy films with various microstructure features were investigated by nanoindentation. With the optimized grain size and microstructure, the film deposited at 350 °C using a power of 100 W exhibits the highest hardness of 11.09 GPa. Our findings not only help understanding the mechanisms during the high entropy film deposition, but also provide guidance in manufacturing other novel high entropy films.

## 1. Introduction

High entropy alloy (HEA) is an emerging class of compositionally complex alloys containing five to thirteen principal metallic elements with a near-equiatomic radio [1,2]. Since HEAs were first reported by Cantor et al. [3], they have attracted great attention in materials science. In fact, the alloy systems could possess high mixing entropy, and therefore, favor the formation of a unique solid solution phase and extraordinary microstructure. Many previous researches have revealed that HEAs have great potential to be used as high temperature materials and structural materials under extreme conditions requiring high hardness, excellent strength and ductility [4,5,6,7]. Furthermore, the large lattice distortion and high configurational mixing entropy of the HEAs offer possibilities for catalytic applications [8,9,10], corrosion resistant material [11] and nuclear applications [12]. HEAs have three forms: bulk, film and powder. Actually, HEA powder is usually applied as catalyst and abradant. Although plenty of researchers focus on the HEA bulk, there are difficulties in controlling the grain size and the uniformity of the phase [13,14].

High entropy film (HEF) is a kind of advanced film with many excellent properties, which is developed based on HEA. HEF not only possesses the superior performance as HEA, but also has various features [15]. The grain size and elementary composition can be easily and accurately controlled in HEF. Additionally, HEF exhibits extraordinarily high yield strengths, one order of magnitude higher than that of its bulk form [5]. HEF provides possibilities and broader room for the application of high entropy materials. Recently, a variety of HEFs have been widely explored. A body-centered cubic (bcc)-structured NbMoTaW HEF exhibits the highest reported strengths and stability for a high-temperature condition, due to the strongly textured and nanometer-sized grains [5]. EI-Atwani et al. combined experiments and modeling to show how a W-based bcc HEF with lamella-like structure leads to exceptional radiation tolerance [16]. TiVCrZrHf HEF with face-centered cubic (fcc) solid solution structure exhibits excellent hardness and modulus.

Among the various kinds of HEF, FeCoNiCrAl is a traditional HEF with good mechanical properties [17] and many researchers have optimized the performance of FeCoNiCrAl HEF by various means [18,19,20]. Magnetron sputtering method is one of the most mature techniques for the preparation of high quality HEF. Deposition parameters, such as substrate temperature, deposition power and type of substrate can play important roles on the microstructure of HFE which could profoundly influence the performance of HEF. Despite intense research on high entropy films, the effects of the deposition parameters on the microstructure of FeCoNiCrAl and the film-forming mechanism are not exhaustively known. Only a few studies involve detailed and systematic investigations of the relationship between the deposition parameters and microstructures. A greater understanding of the parameters’ effects on the structure during the HEF forming process and the impacts of structures on performance is still desired. The detailed understanding could provide guidance for the rational design of new advanced HEFs for future practical applications.

In this study, nano-grain FeCoNiCrAl HEFs with the average grain size ranging from 14 nm to 91 nm were prepared by magnetron sputtering method under different deposition conditions. The objective of this study is to clarify the film-forming mechanism and effects of deposition parameters on the HEF microstructure and elemental uniformity. Nanoindentation was used to characterize the mechanical property of HEFs. The scattering-type scanning near-field optical microscopy (s-SNOM) was introduced as a new approach to illustrate the elemental uniformity of the films in addition to the regular energy-dispersive X-ray spectroscope (EDS) test. To sum up, our study highlights the importance of controlling parameters and provides the strategy to optimize the microstructure and properties in the preparation of HEF.

## 2. Experimental

The FeCoNiCrAl HEFs were deposited by a magnetron sputtering system on three kinds of substrates: Si (100), Si (110) and Si (100) substrate with a SiO_2_ layer on the surface (labeled SiO_2_/Si). The thickness of the SiO_2_ layer is about 300 nm. The targets used in system were alloy target containing FeCoNiCrAl and pure Al target with high purity (99.99%). The base pressure of the chamber was kept lower than 10^−4^ Pa prior to deposition and the argon pressure was 3.8 Pa during sputtering. In order to ensure the homogeneity of the HEF, the sample stage rotated at a speed of 20 RPM. The distance and the angle between target and substrate were 105 mm and 30°, respectively. Table 1 shows the HEF samples prepared under different deposition parameters, including substrate temperature, deposition power and substrate type. Seven experiments with different targets (sample #1), different substrate temperatures (samples #2, #3 and #4), different deposition powers (samples #4 and #5) and different substrate types (samples #4, #6 and #7) were conducted. The deposition powers mentioned above were loaded to the alloy target. To adjust the proportion of elemental composition, HEF sample (sample #1) was prepared by co-sputtering with both a pure Al target and alloy target. The powers of the magnetrons were adjusted to obtain the equal atomic radio of Fe, Co, Ni, Cr and Al in preparation of sample #1.

The microstructure of the as-made HEFs was characterized by transmission electron microscopy (TEM) using a Tecnai F30 TEM (FEI, Hillsboro, OH, USA). The morphology and the thickness of the samples were characterized by plan-view and cross-sectional scanning electron microscope (SEM, FEI, Hillsboro, OH, USA). The grain size was statistically analyzed by a program, Nanomeasure. The measurements of elemental composition of the films were performed by EDS on an FEI Nano SEM 430 and a Tecnai F20 TEM. Phases of the films were identified by X-ray diffraction (XRD) using a Cu Kα radiation on a Panalytical Empyrean (PANalytical, Almelo, The Netherlands). Scattering-type scanning near-field optical microscopy (s-SNOM) was used to further characterize the surfaces of the HEF samples. The system used in the investigations was a Neaspec NeaSNOM Microscope (Neaspec, Munich, Germany) with an excitation wavelength of 1550 nm. The atomic force microscopy (AFM) probe was a Mikromasch Hq:NSC19/Cr-Au, which has a resonance frequency of 65 kHz, force constant of 0.5 N/m and tip radius of less than 35 nm. Nanoindentation measurements were conducted to evaluate the hardness of the HEFs using Agilent Nano Indenter G200 (Agilent, Beijing, China).

## 3. Results and Discussion

### 3.1. Growth Mechanism of the HEF

**Figure 1:**
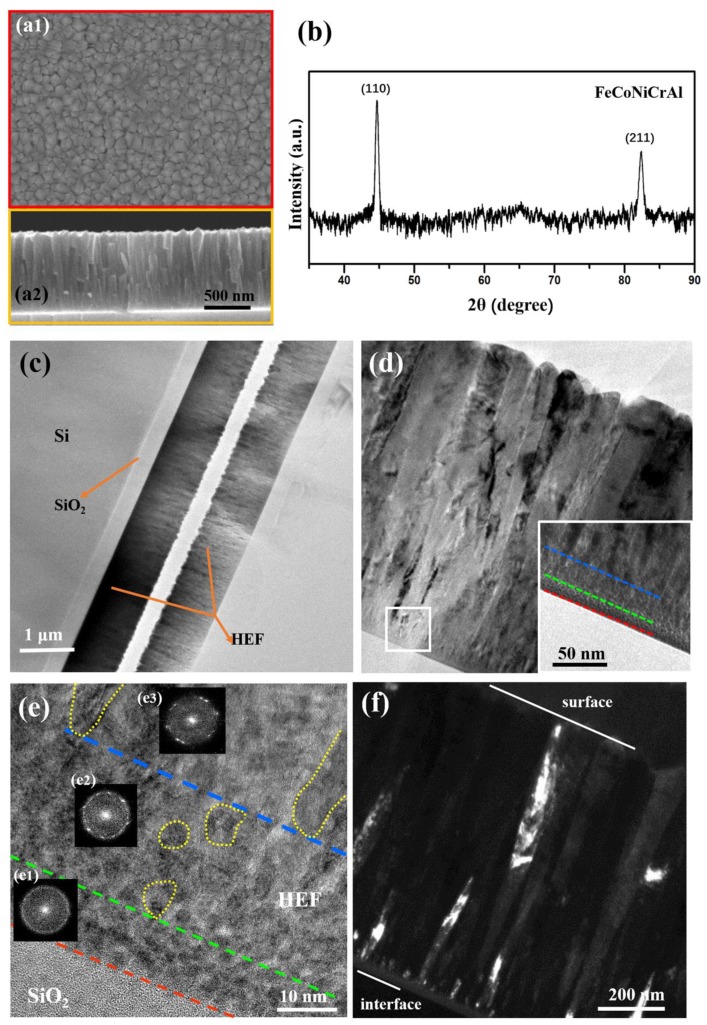
(**a**) SEM images of HEF from (**a1**) plan-view and (**a2**) cross-sectional view. (**b**) XRD pattern of HEF showing the (110) and (211) plane with body-centered cubic (bcc) structure. (**c**) Cross-sectional TEM image with low magnification showing the full view of the sample. (**d**) High-magnification TEM images of HEF, and inset is the enlarged image of the white box area. The regions with different structures are separated by dotted lines. (**e**) HRTEM image of the selected area in (**d**) showing the detailed microstructure of the three typical regions. Insets are the corresponding FFT patterns. Some of the nanograins and columnar grains are circled by yellow dotted lines. (**f**) Dark-field TEM image of HEF. The surface of the film and interface between film and substrate are marked in the image.

The HEF (sample #1) was prepared by magnetron co-sputtering deposition system with an alloy target and a pure Al target. Figure 1 shows the results of the microstructure and phase of the sample. The SEM images of film surface and internal cross-section are shown in Figure 1(a1,a2), indicating that the sample consists of irregularly-shaped columnar grains. The in-plane grains have polygon morphology and the statistical grain size is 92 ± 26 nm. The columnar crystal structure is confirmed by the cross-sectional image and the top of the columnar grains has angle shape. The thickness of the film is 978 ± 5 nm. Figure 1b shows the XRD pattern of the HEF. Grazing incidence mode was used in the XRD measurement to avoid the interference of the substrate signal. The diffraction peaks located at 44.6° and 82.3° indicate that the FeCoNiCrAl phase with bcc structure is formed.

A cross-sectional TEM image of the HEF (Figure 1c) shows that the film was deposited on the SiO_2_/Si substrate and the thickness of the SiO_2_ is about 300 nm. Figure 1d is the enlarged image of the film and the thickness of the film measured from TEM image is 980 ± 4 nm, which is consistent with the result of SEM image. Most of the grains exhibit a columnar structure in the sample and the width of a columnar grain is related to the location. The portion of columnar grain close to the surface is wider than that close to the substrate, hence the shape of the columnar grain is like a ladder from the cross-sectional view. Besides, the partial area which is very close to the substrate has a special microstructure. The top-right inset is the enlarged image of the selected area (white box) in Figure 1d and clearly shows the difference from columnar crystal structure. Amorphous structure (located between red line and green line) and equiaxial nanocrystalline structure (located between green line and blue line) exist in this area, and columnar crystal forms above the nanocrystalline region. The width of the amorphous region and nanocrystalline region is approximately 8 nm and 20 nm respectively.

Figure 1e is the high-resolution TEM (HRTEM) image of the region very close to the substrate, and the insets (Figure 1(e1–e3)) are the fast Fourier transform (FFT) patterns of the corresponding area. Some domains with sizes of about 2 nm exist in the region next to the substrate and the atomic arrangement is disordered in the domains. The FFT shows the amorphous holes without diffraction points, which demonstrate the amorphous structure in this region. Above the amorphous region, some equiaxial nanograins (labeled as a yellow dotted line between a green dotted line and a blue dotted line) are observed and the FFT pattern with a polycrystalline ring also confirmed the nanocrystalline structure. Figure 1(e3) is the FFT pattern of the area above the nanocrystalline region, which shows the diffraction points clearly. The grain shape tends to be columnar and the grain size becomes larger (labeled by a yellow dotted line above the blue dotted line). The closer to the film surface, the larger the columnar crystal grain size. Figure 1f is dark-field TEM image of the film, and the bright areas represent grains with the same orientation. A region with gray contrast existing above the interface, between substrate and HEF, indicates the amorphous structure. Additionally, a lot of bright areas with small sizes represent the equiaxial nanograins. Above the nanocrystalline region, some large bright areas indicate the existence of columnar grains. The regions containing amorphous domains and nanocrystalline structure were also observed in other samples which are not shown here.

During the film deposition process, the atoms on the target surface were sputtered by high-energy argon ions and then were deposited onto the substrate under the effect of a dual function of magnetic and electric fields. The process of coating atoms depositing onto the substrate to form the thin film occurs in three steps: nucleation, islands growth and coalescence, and grain coarsening [21,22]. For the HEF, the sputtered atoms with energy interact with the surface atoms of substrate, and become loosely-bonded adatoms by transferring kinetic energy. The island structure forms by the adatoms’ aggregation and diffusion. As the number of sputtered atoms increases, the island coalesces to be a small domain under the driving force of atomic diffusion. The adatoms are composed of five kinds of atoms with different atomic radii, leading to the difficulty to form ordered crystal islands in the initial stage. Hence, the structure of the region near the substrate is amorphous.

After the amorphous domain formation, the sputtered atoms would exchange energy with the high entropy domain rather than substrate atoms. The domains support some sites with low energy to make the sputtered atoms grow extendedly, and then the nanograins with small grain size form. As the continued deposition of energetic atoms, film thickening proceeds through local epitaxy on the individual grains to form columnar grains, accompanying the recrystallization and atoms’ surface migration. In the whole process, the energy of sputtered atoms and the energy of deposited atoms on the substrates are the key factors affecting the film formation.

### 3.2. The Effects of Deposition Parameters on Microstructure and Elemental Uniformity

The power loaded on the target and the substrate temperature determine the energy of sputtered atoms and deposited atoms, respectively. Besides, the crystal orientation of substrate would affect the island formation during the film deposition. Therefore, the power, substrate temperature and the type of substrate are three important parameters that could affect the phase and microstructure of HEF. FeCoNiCrAl HEFs with different deposition conditions were prepared (summarized in the Table 1) to investigate the effects of the parameters.

**Figure 2:**
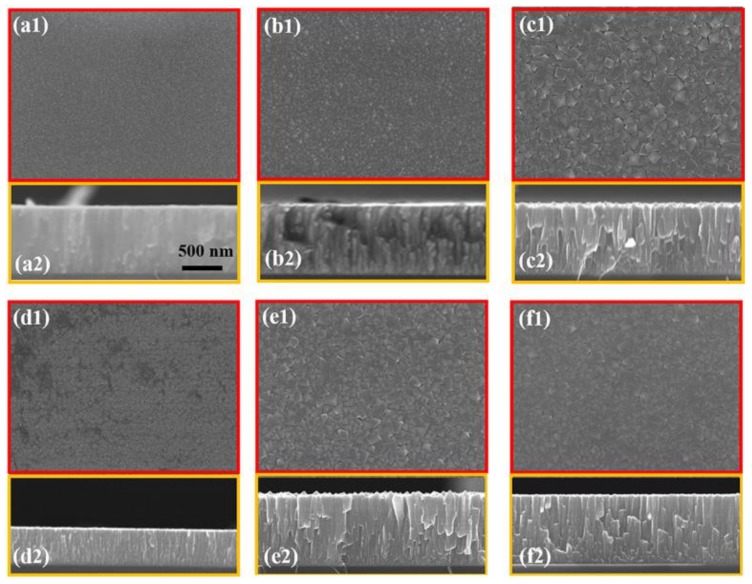
SEM images of HEF deposited with different parameters. Plan-view images for (**a1**) RT, 300 W, on Si (100); (**b1**) 250 °C, 300 W, on Si (100); (**c1**) 350 °C, 300 W, on Si (100); (**d1**) 350 °C, 100 W, on Si (100); (**e1**) 350 °C, 300 W, on Si (110); (**f1**) 350 °C, 300 W, on SiO_2_/Si. (**a2**–**f2**) are the corresponding cross-sectional images.

Figure 2 is the SEM images of HEF samples with different deposition parameters, including plan-view images (Figure 2(a1–f1)) and corresponding cross-sectional images (Figure 2(a2–f2)). All of the images have the same scale bar, as marked in Figure 2(a2). Figure 2a–c represent the images of the HEF samples deposited on substrate of Si (100) with the power of 300 W. The substrate temperatures (T) are room temperature (RT), 250 °C and 350 °C respectively. The surface of these samples is intact without holes or cracks, and the thickness of the films is about 975 ± 5 nm.

The edge profile of the grain is smooth when the substrate temperature is RT, as shown in Figure 2(a1). By counting the grain size from the plan-view SEM image, the statistical grain size of the columnar crystals close to the surface is 14 ± 3 nm. The characteristic of columnar crystal is not obvious from the cross-sectional view (Figure 2(a2)). When T = 250 °C, the sample grain size increases to 38 ± 10 nm (Figure 2(b1)), and the anisotropic grains composed of small crystal grains are observed, as shown in Figure 2(b2). When T = 350 °C, Figure 2(c1) exhibits the irregularly-shaped grains with an average size of 91 ± 28 nm, and the obvious columnar crystal structure occurs (Figure 2(c2)). This indicates that as the substrate temperature increases, the grain shape changes from round to polygon with the increase of the grain size.

Figure 2d illustrates the HEF sample deposited on Si (100) with the substrate temperature of 350 °C, and the power reduced to 100 W. Compared with sample #4 deposited with power of 300 W (as shown in Figure 2c), the statistic grain size of this sample decreases to 25 ± 6 nm and the edge profile of the crystal is more rounded (Figure 2(d1)). This means the power is a significant factor affecting the grain size of columnar crystal. The characteristic columnar crystals are observed in Figure 2(d2). The film with the thickness of 247 ± 4 nm is thinner than other films due to the lower deposition rate.

Figure 2e,f show the SEM images of the samples, which were deposited on substrates of Si (110) and SiO_2_/Si, respectively, and the power was 300 W with T = 350 °C. These two samples had little difference in their morphologies with the sample #4 which was deposited on substrate of Si (100) with a power of 300 W and substrate temperature of 350 °C. The statistical grain sizes are 89 ± 26 nm and 86 ± 23 nm, respectively, which are also close to the grain size of the sample #4. The results suggest that the structure of HEFs is almost unaffected by the crystal orientation of substrate.

**Figure 3:**
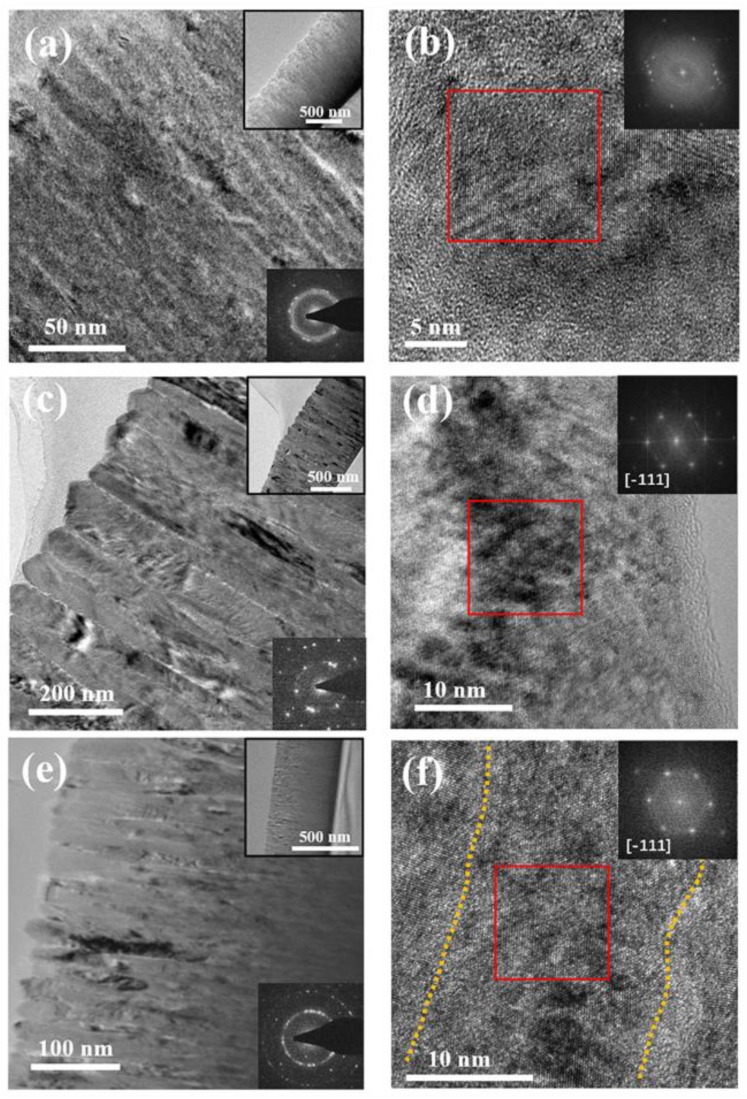
(**a**) TEM and (**b**) HRTEM images of HEF deposited on Si(100) at RT with a power of 300 W; (**c**) TEM and (**d**) HRTEM images of HEF deposited at 350 °C with a power of 300 W; (**e**) TEM and (**f**) HRTEM images of HEF deposited at 350 °C with a power of 100 W. The top-right insets are the low-magnification images and bottom-right insets are SAED patterns in (**a**), (**c**) and (**e**), respectively. The top-right insets are FFT patterns of the selected area (red box) in (**b**), (**d**) and (**f**), respectively. In figure (**f**), the grain boundaries are marked by dotted lines.

The substrate temperature and the power significantly affect the morphology and grain size of HEF. A microstructure at the atomic scale is necessary to explore. Figure 3 illustrates the detailed microstructure of HEF samples #2, #4 and #5, which were all deposited on the substrate of Si (100). Figure 3a represents the TEM image of HEF with the substrate temperature of RT and the power of 300 W. The top-right inset is the low-magnification image to demonstrate the overall condition of the film. The grain boundary of the sample cannot be clearly observed. The bottom-right inset is the selected area electron diffraction (SAED) pattern of the sample. It exhibits amorphous holes, except for diffraction points, indicating that crystal structures together with amorphous structures exist in the film. The HRTEM image shown in Figure 3b demonstrates the nanocrystal with small grain size and the disordered structure in some areas. The corresponding FFT pattern (top-right inset in Figure 3b) shows multiple crystal orientations, indicating the polycrystalline structure. All of the results confirm that the crystallinity of the HEF sample deposited at room temperature is not good enough. What happens under a higher substrate temperature?

Figure 3c,d clearly shows the microstructure of HEF samples deposited at the substrate temperature of 350 °C. The depth of the columnar crystal is clearly observed in Figure 3c and the average width of columnar grain is about 82 nm. The high aspect-radio of the columnar grains results from the anisotropy growth velocity under deposition [23]. The edge profile of the columnar grain near the surface is choppy, resulting from the wide angular distribution of the deposition flux and atomic shadowing [21]. The SAED pattern (bottom-right inset in Figure 3c) shows the diffraction points without amorphous holes, implying that the sample has a relatively complete crystal structure. Figure 3d is an HRTEM image of one columnar grain in Figure 3c, illustrating the ordered lattice arrangement. The FFT pattern (top-right inset in Figure 3d) of the red box area indicates that crystal orientation of this grain is [−111]. Combining with the measured lattice spacing (0.208 nm), it can be clarified that FeCoNiCrAl phase with bcc structure is formed in the film.

Figure 3e,f show the TEM images of HEF sample deposited at a substrate temperature of 350 °C with a power of 100 W. The columnar crystal structure could also be observed and the average width of the columnar grain is about 22 nm, which is smaller than that of the sample deposited using a power of 300 W. Besides, most columnar grains are closely arranged together and no distinct gaps or intra-columnar voids exist. The polycrystalline ring illustrated in the SAED pattern (bottom-right inset in Figure 3e) indicates that the polycrystalline structure with a small grain size has more complicated crystal orientations. Detailed crystal structure is characterized by HRTEM (Figure 3f), which is similar to the sample deposited with power of 300 W. FFT pattern (top-right inset in Figure 3f) of the red box area indicates that the crystal orientation is [−111] and the columnar grain has a perfect single crystal structure. Meanwhile, the grain boundaries between columnar grains are clearly observed, marked by the dotted line. The edge profile of the columnar grain is more smooth than that of sample #4. These results clarify that HEF sample deposited using a power of 100 W with the substrate temperature of 350 °C has a good-crystallinity structure with small grain size.

The microstructure of the HEF deposited at room temperature is a nanocrystalline structure with some disordered structure, and no distinct columnar crystal structure is observed. In contrast, all of the films deposited at higher substrate temperature have a good crystallinity without amorphous structure. During the deposition of films, the energy of deposited adatoms affects the ability and rate of migration, which would be determined by the substrate temperature. The sputtered atoms interact with the atoms on the substrate at room temperature and become the adatoms by exchanging energy. Then, some adatoms are captured at low-energy lattice sites to form nanocrystals [22]. When the low-energy lattice sites are filled up, the remaining adatoms do not have enough energy to migrate to the stable lattice sites, and the disordered structure forms by these adatoms. The higher substrate temperature provides energy for the deposited adatoms to support their migration to form a continuous crystal structure. As a result, the crystal quality could be improved by increasing the substrate temperature.

After the crystal structure forms in the HEF, the growth of the grain is controlled by two processes: adatom surface diffusion and recrystallization through grain boundary migration. The power of the target provides energy for sputtered atoms to interact with atoms on the substrate. The energy of sputtered atom is obtained from the energy exchange with positive ions, which is dependent on the electric field and magnetic field controlled by power. Higher power would increase the kinetic energy and the flux of sputtered atoms, which makes the surface diffusion of adatoms easier [21], resulting in the growth of grain size. The recrystallization through grain boundary migration is affected by the substrate temperature. The grain size increases with increasing substrate temperature because of the enhanced thermal migration of grain boundary under deposition [24,25]. Hence, reducing the substrate temperature and power provides the opportunity to decrease the grain size. Nevertheless, the lower substrate temperature could cause the poor crystallinity. Therefore, the deposition condition of higher substrate temperature and lower power can be used to prepare HEF samples with small grain size and high crystalline quality. In general, the crystal orientation of the substrate would affect the crystallinity of the film by generating stress when the island forms. However, the structure formed in the initial stage is an amorphous domain in the HEF sample, which is less affected by the stress. As a result, the microstructure of HEFs is almost unaffected by the crystal orientation of the substrate.

**Figure 4:**
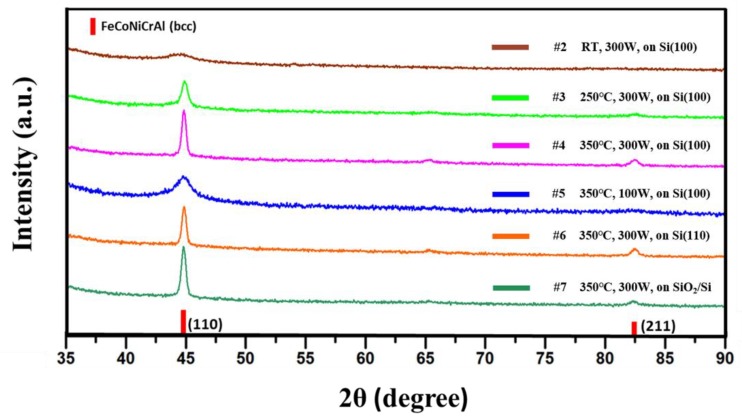
XRD patterns of the HEF samples deposited with different parameters.

To further reveal the effect of the key parameters on phase composition of HEF, XRD experiments were conducted in grazing incidence mode. Figure 4 illustrates the XRD patterns of the HEF samples prepared with different parameters. All of the spectrums emerge an easy-to-observe peak, which has the position of 44.6°, indicating the formation of bcc-structured FeCoNiCrAl phase with (110) plane in the film. Besides, samples #3, #4, #6 and #7 also have a small peak at the position of 82.3° representing the bcc-structure FeCoNiCrAl phase with a (211) plane. The XRD results illustrate that all the film samples have a preferred orientation of [110] with single bcc structure, which is different from the FeCoNiCrAl high entropy alloys prepared by other methods having mixed phase structures [26,27]. To further examine the average grain size of axial columnar crystal, the full width at half maximum (FWHM) of the main peak is given in Table 2, and the average grain size is calculated using Scherrer formula

D = K·λ/B·cosθ

where the Scherrer constant K = 0.89, wavelength λ = 0.154 nm and B = FWHM. The results of samples #2, #3 and #4 illustrate that high substrate temperature would promote the growth of crystal size. Besides, the low power results in smaller grain size of the columnar crystal by comparing the results of samples #4 and #5. Meanwhile, the type of substrate has no significant effect when analyzing the results of samples #4, #6 and #7, which is consistent with previous results.

**Figure 5:**
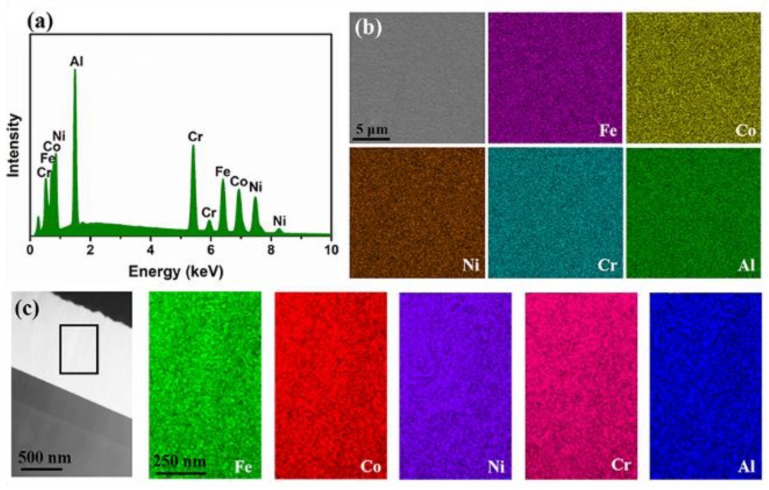
(**a**) SEM-EDS spectroscopy of HEF showing that the atomic radio (Fe:Co:Ni:Cr:Al) is 19.4:19.5:20.5:20.0:20.6. (**b**) SEM image from plan-view and the corresponding SEM-EDS element mapping of the HEF. (**c**) STEM image from cross-sectional view and the corresponding STEM-EDS element mapping.

To characterize the chemical composition of the HEF samples, EDS measurements were carried out to explore the atomic ratio and distribution of the elements. Figure 5 shows the SEM-EDS results and STEM-EDS results of HEF sample #1, illustrating the elemental distribution from plan-view and cross-sectional view, respectively. The spectroscopy (Figure 5a) indicates that the film consists of five elements (Fe, Co, Ni, Cr and Al) in a near-equal atomic percentage (19.4:19.5:20.5:20.0:20.6) without obvious difference between the solute and solvent. The elemental distribution in plan-view was characterized by SEM-EDS (Figure 5b) and the results show that the five elements are uniformly distributed without segregation. The EDS mapping with higher magnification was conducted, and the result is consistent. Figure 5c shows the STEM image of the film from a cross-sectional view and the EDS mapping of the selected area, confirming that the HEF sample has a random distribution of the five elements in the cross-sectional view. The similar results are observed in other HEF samples (not shown here). The chemical composition uniformity of the sample prepared by magnetron sputtering is better than other techniques, such as art melting and hot compression [27,28,29], benefiting from the process of sputtering and deposition at the atomic scale [30].

EDS is a common method that could be used to characterize the chemical composition of materials [29,31]. Nevertheless, the depth that EDS detects reaches hundreds of nanometers. This means the results of EDS represent an average over the range of the certain depth. In order to investigate the elemental uniformity of the sample surface clearly, a novel method was introduced to the characterization of HEFs. Scattering-type scanning near-field optical microscopy (s-SNOM) has resolved the optical diffraction limit and exhibits the ability of imaging with nano-scale resolution [32]. Usually, it is built as a bimodal tool comprising both s-SNOM and AFM. In a common setting, the tip of a metal-coated AFM probe is illuminated by a laser beam during the sample-scanning and the scattered light from the tip-sample interaction volume is detected using an interferometric detection scheme for suppressing the background light. The setting permits the acquisition of both AFM and s-SNOM images simultaneously. The most preferred interferometric detection scheme is known as “pseudo-heterodyne” [33] using a modified Michelson interferometer, allowing for simultaneous acquisition of both amplitude and phase images of the scattered light.

**Figure 6:**
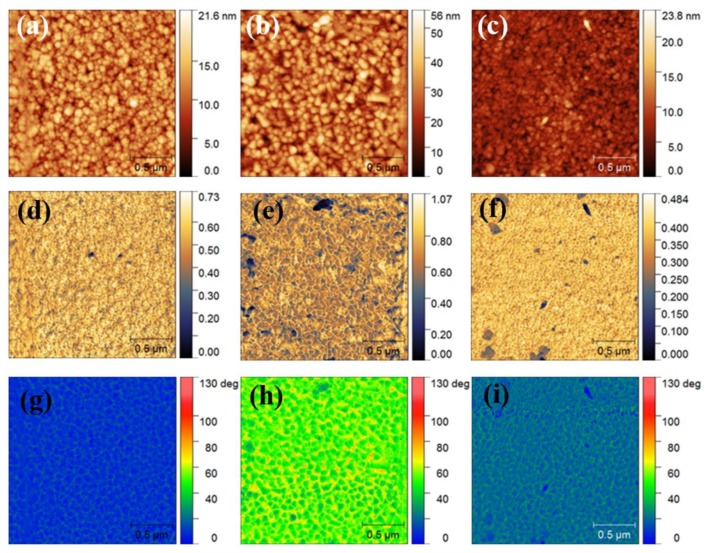
AFM images of HEF deposited on Si (100) at (**a**) RT, 300 W; (**b**) 350 °C, 300 W; (**c**) 350 °C, 100 W. The s-SNOM amplitude images of HEF deposited at (**d**) RT, 300 W; (**e**) 350 °C, 300 W; (**f**) 350 °C, 100 W. The s-SNOM phase images of HEF deposited at (**g**) RT, 300 W; (**h**) 350 °C, 300 W; (**i**) 350 °C, 100 W.

Figure 6 shows the AFM images (Figure 6a–c), s-SNOM amplitude images (Figure 6d–f) and phase images (Figure 6g–i) of the HEF samples #2, #4 and #5, respectively. While the AFM images reveal the topography of the sample surface, the s-SNOM images (amplitude and phase) reveal the optical properties of the samples. According to the general conception, the amplitude signal is linked to the dispersion, while the phase signal is linked to the optical absorption [34,35]. The surfaces of HEF samples deposited at room temperature with the power of 300 W (sample #2) and at 350 °C with the power of 100 W (sample #5) have similar morphologies, which consist of round particles of small sizes (Figure 6a,c). In contrast, polygonal-shaped features appear on the surface of HEF sample deposited at 350 °C with the power of 300 W (sample #4), as shown in Figure 6b. The HEF sample #4 has the roughest surface because of the large grain size. Samples #2 and #5 have similar amplitudes and phase images (Figure 6d–g,i), indicating the dispersion and absorption properties of the HEF samples are similar. Meanwhile, the amplitude and phase SNOM images of samples #4 are obviously different, shown in Figure 6e,h. In general, the chemical composition and the surface topography could affect the optical properties of the sample, hence resulting in SNOM images. The larger grain size and surface roughness of sample #4 results in the obvious difference in its SNOM images compared with the images of other two samples.

In order to explain the results quantitatively, the entropy calculation which reflects the uniformity of chemical composition was conducted by using the s-SNOM data. The Shannon information entropy and the gray-level co-occurrence (GLCM) entropy were calculated. The Shannon information entropy and GLCM entropy are used to investigate the uniformity of element distribution. Set the discrete variate *X* having *n* possible values (*x*_1_, *x*_2_, …, *x*_n_) and each value of probability is [*p*(*x*_1_), *p*(*x*_2_), …, *p*(*x*_n_)]. The Shannon information entropy of X is defined as follows:

$$E\left(X\right)=-\overset{n}{\underset{i=1}{\sum }}p\left(x_{i}\right)\cdot \log\left[p\left(x_{i}\right)\right]$$

In the case of an image, the discrete variates *X* are the pixel values, while *p*(*x_i_*) is the probability of occurrence of a pixel value *x_i_*. The calculated Shannon information entropies are 6.78 ± 0.28, 7.27 ± 0.22 and 6.91 ± 0.15 for samples #2, #4 and #5, respectively. The GLCM matrix is a second-order statistical method which provides information on the spatial relationships between intensities of the pixels in a given image. The entropy is calculated with the GLCM texture plugin in the software of ImageJ. The GLCM is constructed by counting the number of occurrences of a gray level adjacent to another gray level, at a specified pixel distance. Each result is divided by the total number of elements to obtain a probability. The matrix elements are the probability of the gray level co-occurrence between pixels, with the rows and columns of the matrix representing the gray levels in the image. The matrix can be computed for adjacent pixel either in horizontal (0°), vertical (90°) or diagonal (45°, 135°) direction. In our case, average values for the four directions were considered for the computed parameters. Thus, we proposed to calculate the entropy more precisely by examining the mentioned organization parameters.

The calculated results inform that the GLCM entropy of the sample #4 was the largest, which was 8.70 ± 0.22. The samples #2 and #5 had GLCM entropies of 8.51 ± 0.24 and 8.60 ± 0.22, respectively. Higher entropy suggests a higher disorder, indicating the better uniformity of element distribution. Combining the results of Shannon entropy, we can conclude that the chemical composition distribution of the HEF deposited at 350 °C using a power of 300 W is more uniform than that of HEF deposited at room temperature and that of HEF deposited using a power of 100 W. The deposited adatoms get more energy in the substrate with a high temperature, resulting in the faster migration rate in the HEF. On the other hand, the power determines the movement of sputtered atoms, and increasing the power could effectively increase the interaction energy. Therefore, the elemental uniformity can be optimized by using high substrate temperature and power during the film deposition.

### 3.3. Mechanical Property of HEFs with Different Microstructures

**Figure 7:**
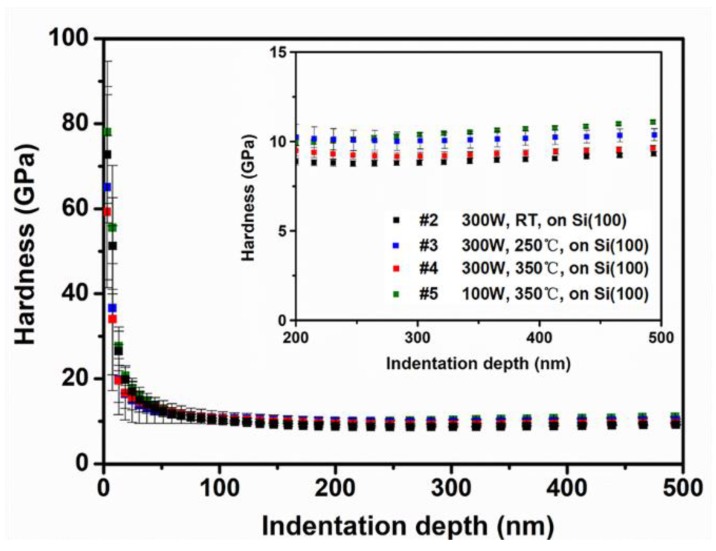
The hardness of HEF samples versus indentation depth. and the inset is the enlarged profile of the flat region.

The hardness of the HEFs deposited with different experimental parameters was measured by nanoindentation with continuous stiffness mode (CMS). The hardness versus indentation depth curves of the films are shown in Figure 7. The hardness values of depths ranging from 0 nm to 500 nm were detected. As can be seen in Figure 7, the profile of the hardness can be mainly divided into two ranges. When the depth is less than 100 nm, the hardness decreases with the increase of indentation depth, rapidly. This phenomenon can be interpreted as indentation size effect (ISE) which is common in the crystalline materials [36]. When the depth is in the range of 100–500 nm, an almost flat profile is obtained and the hardness values of the samples are extracted in the flat region [37]. The enlarged profile of the indentation depth ranging from 200 nm to 500 nm is shown in the top-right inset to observe the difference in hardness of the samples clearly. The hardness of HEF sample #5 deposited at 350 °C using a power of 100 W is the highest with the value of 11.09 GPa. The hardness of samples #2, #3 and #4, which were deposited at different substrate temperatures (RT, 250 °C and 350 °C, respectively) with the same power (300 W), are 9.33 GPa, 10.37 GPa and 9.63 GPa, respectively. Besides, the hardness of samples #6 and #7 which were deposited on the substrate of Si (110) and SiO_2_/Si are 9.66 GPa and 9.59 GPa (not shown here), and both of them are close to the hardness of the sample #4.

In general, the hardness is influenced by the microstructure and the grain size in the film materials. The HEF deposited at the substrate temperature of 350 °C a using power of 100 W (sample #5) has a small grain size with columnar crystalline structure, which was clarified by the results of XRD and TEM. This means a high volume fraction of grain boundary exists in the film. Grain boundary plays an important role in preventing the slip of dislocations in the films, and thus the hardness of HEF with a larger grain boundary density should be larger [37,38]. As the grain size of samples #3 and #4 is larger than that of sample #5, the density of grain boundary is less, leading to the lower hardness. Sample #2 has the smallest grain size according to the XRD results, however, the hardness of the sample is the lowest. Microstructure of the film is also an important factor for influencing the mechanical properties. The sample #2 has nanocrystalline together with amorphous structure, rather than the columnar structure. The poor crystallinity leads to the decrease of obstacles for the slip of dislocation, thus the hardness of the sample #2 is lower than that of other samples.

## 4. Conclusions

High entropy films containing five elements (Fe, Co, Ni, Cr, and Al) with a near-equal atomic percentages were successfully synthesized by magnetron sputtering with different parameters. The microstructure of the samples was analyzed in detail to clarify the HEF growth mechanism. Firstly, the sputtered atoms interact with the substrate atoms and become adatoms, and then the amorphous domain comes to exist on the substrate by the migration and aggregation of adatoms. Secondly, an equiaxial nanocrystalline structure is formed above the amorphous domain with the deposition of energy. Finally, film thickening proceeds through local epitaxy on the individual grains to form columnar crystalline structure. The microstructure in terms of grain shape and size and the crystallinity in HEFs is revealed in this study. The substrate temperature and the power loaded on the target significantly affect the crystal structure and size by controlling the movement and diffusion of sputtered atoms and adatoms. The orientation of the substrate has a less obvious effect on microstructure due to the amorphous domain existing in the initial stage of HEF formation. The s-SNOM, as a novel approach, was introduced to characterize the elemental uniformity in addition to the regular EDS test. It is found that HEFs deposited with both a higher substrate temperature and power have better elemental uniformity, by calculating the Shannon entropy and GLCM entropy. An HEF sample deposited at 350 °C with power of 100 W shows the best property, with the hardness of 11.09 GPa, resulting from the high density of grain boundary obstructing dislocation slip and the good crystallinity.

## Figures and Tables

**Table 1:** The deposition parameters for high entropy film (HEF) samples.

Sample Number	Deposition Parameters		
	Power (W)	Substrate Temperature (℃)	Substrate
#1	300 _alloy target_ and 20 _Al target_	350	SiO_2_/Si
#2	300	RT	Si (100)
#3	300	250	Si (100)
#4	300	350	Si (100)
#5	100	350	Si (100)
#6	300	350	Si (110)
#7	300	350	SiO_2_/Si

**Table 2:** Full width at half maximum (FWHM) of the diffraction peak of the (110) plane and the corresponding calculated grain size for the HEFs deposited with different parameters.

Sample Number	Deposition Parameters			FWHM (°)	Grain Size (nm)
	Power (W)	Substrate Temperature (℃)	Substrate		
#2	300	RT	Si (100)	2.54	3.3
#3	300	250	Si (100)	0.53	16
#4	300	350	Si (100)	0.32	27
#5	100	350	Si (100)	1.64	5.2
#6	300	350	Si (110)	0.33	26
#7	300	350	SiO_2_/Si	0.32	27
